# Current Drug Development Pipeline for MASLD and MASH: Focusing on Cardiovascular Comorbidities

**DOI:** 10.3390/biomedicines14040909

**Published:** 2026-04-16

**Authors:** Veronika A. Prikhodko, Sergey V. Okovityi

**Affiliations:** 1 Department of Pharmacology and Clinical Pharmacology, Saint Petersburg State Chemical and Pharmaceutical University, 14A Prof. Popov Str., 197022 St. Petersburg, Russia; sergey.okovity@pharminnotech.com; 2 Scientific, Clinical and Educational Center of Gastroenterology and Hepatology, Saint Petersburg State University, 7/9 Universitetskaya Emb., 199034 St. Petersburg, Russia

**Keywords:** metabolic dysfunction-associated steatotic liver disease, metabolic dysfunction-associated steatohepatitis, cardiovascular disease, clinical trials, drug development

## Abstract

Metabolic dysfunction-associated steatotic liver disease (MASLD) is characterized by an exceptionally high global prevalence that is projected to continue rising in the near future. MASLD is strongly associated with a spectrum of cardiometabolic risk factors, and may itself, in turn, contribute to cardiovascular morbidity and mortality. This interconnection warrants the development of integrated treatment strategies targeting shared pathophysiological processes and addressing both hepatic, metabolic, and cardiovascular outcomes. In this work, we review the modern MASLD clinical development pipeline and highlight the most prominent drug candidates with known or purported cardiovascular benefits, discussing mechanistic links and supporting evidence ranging from preclinical experiments to real-world data. Although the drug development pipeline is extensive and diverse, evidence supporting cardiovascular benefits for most candidate molecules remains limited. Both of the FDA-approved therapies, resmetirom and semaglutide, have been found to significantly reduce the risk of major adverse cardiovascular events as well as cardiovascular and all-cause mortality in patients with MASH. In addition, significant improvements were observed in patients with heart failure with preserved ejection fraction treated with semaglutide, highlighting incretin mimetics as a promising class for managing cardiovascular disease concomitant with MASLD/MASH. Other investigational compounds, targeting the farnesoid X receptor, peroxisome proliferator-activated receptors, de novo lipogenesis enzymes, and fibroblast growth factors, have demonstrated improvements in blood lipid spectrum and glycemic control; however, their clinical effectiveness in patients at cardiovascular risk has yet to be established.

## 1. Introduction

Metabolic dysfunction-associated steatotic liver disease (MASLD) is the most common cause of chronic liver disease worldwide, affecting more than 38% of adults [1] and 7% to 14% of children [2]. By 2040, the global prevalence of MASLD in adults is projected to exceed 50% [3]. The spectrum of MASLD includes steatosis, metabolic dysfunction-associated steatohepatitis (MASH), fibrosis, cirrhosis and MASH-related hepatocellular carcinoma [4]. The term ‘MASLD’ was introduced in 2023 to reflect the importance of the association between hepatic steatosis and cardiometabolic risk factors, including dysglycemia or type 2 diabetes mellitus (T2DM), overweight/obesity, arterial hypertension, and atherogenic dyslipidemia, specifically hypertriglyceridemia and/or low high-density lipoprotein cholesterol (HDL-C) [4,5].

The etiopathogenesis of MASLD is complex and multifactorial, and can be explained by the “multiple hits” hypothesis, in which several parallel insults drive disease initiation and progression [6,7]. Genetic and epigenetic susceptibility factors, gut dysbiosis, dietary habits, unhealthy lifestyle, and overweight/obesity contribute to the development of insulin resistance, hepatic steatosis, lipotoxicity, and systemic low-grade inflammation. These processes cause oxidative and endoplasmic reticulum stress in hepatocytes, leading to an impairment of autophagy and apoptosis and further promoting localized inflammation. Ultimately, a vicious cycle is initiated, characterized by hepatocyte death, immune cell attraction, and activation of hepatic stellate cells, resulting in liver fibrosis [6,7,8].

A large body of evidence indicates that MASLD and MASH patients are at increased risk for subclinical cardiovascular disease (CVD) as well as for major adverse cardiovascular events (MACE), which are the predominant cause of death in this population, even before the onset of advanced liver disease [9,10]. The main shared pathophysiological mechanisms include oxidative and endoplasmic reticulum stress, systemic and hepatic insulin resistance, low-grade inflammation, and endothelial dysfunction. All of these phenomena contribute to the development of atherosclerosis, vascular inflammation and remodeling, endothelial damage, hypercoagulability, and cardiac electrophysiological abnormalities [10].

Lifestyle modifications, particularly weight loss, dietary changes, physical exercise, and alcohol abstinence, remain the cornerstone of MASLD treatment [4]. However, given the multisystem nature of MASLD, pharmacotherapy is increasingly recognized as essential not only to attenuate the progression of hepatic injury but also to address coexisting cardiometabolic comorbidities [11]. Despite the high and growing global burden of MASLD/MASH, only two therapies, resmetirom and semaglutide, are currently approved by the FDA [12]. Both agents primarily target patients with F2-F3 liver fibrosis, whereas treating simple hepatic steatosis and associated metabolic dysfunction remains a largely unmet clinical need. This paper provides a comprehensive review of the current clinical drug development pipeline, with particular emphasis on emerging therapeutic strategies aimed at reducing cardiovascular risk in MASLD and MASH.

## 2. Methods

This work provides a narrative literature overview synthesizing the findings on cardiovascular effects of molecules that are in active clinical development for MASLD and/or MASH. Preclinical stage candidates, approved therapies and investigational drugs not having an active indication of MASLD/MASH fall outside the scope of the present review and are not discussed below.

In February 2026, we conducted a search across several databases, including PubMed (MEDLINE), Europe PMC, Cochrane Database of Systematic Reviews, Google Scholar, clinical trial registers of the United States, European Union, and the United Kingdom, as well as other publicly available sources. The relevant literature was identified via search terms such as “metabolic dysfunction-associated steatotic liver disease”, “metabolic dysfunction-associated steatohepatitis”, “cardiovascular event”, “cardiovascular risk”, “dyslipidemia”, etc.

Compound classes for which clinical trial data on cardiovascular events or surrogate cardiometabolic markers in patients with MASLD/MASH were available were included in the review. Preclinical findings were not prioritized and were discussed only to provide additional context where clinical data were limited. The clinical trial status and development stage of each drug candidate were verified based on data from public clinical trial registers and official developer websites.

## 3. THRβ Agonists

In 2024, resmetirom (Rezdiffra^®^, MGL-3196) became the first thyroid hormone receptor β (THRβ) partial agonist to receive FDA and EMA approval for the treatment of noncirrhotic MASH with moderate-to-advanced fibrosis in adults. Selective activation of hepatic THRβ induces a state of so-called ‘functional hyperthyroidism’, promoting mitochondrial biogenesis and mitophagy, lipophagy, fatty acid uptake and β-oxidation, and lipoprotein turnover. THRβ upregulation plays an important role in reducing the hepatic lipid burden as well as systemic levels of triglycerides (TGs), cholesterol, and free fatty acids [13,14]. Given the modality of their action, selective THRβ agonists represent a promising therapeutic approach for atherogenic dyslipidemia accompanying MASLD and MASH.

In one Phase 2b and two Phase 3 randomized, placebo-controlled trials, resmetirom treatment (80 or 100 mg once daily (QD)) resulted in significant resolution of steatohepatitis and fibrosis according to pathology and non-invasive tests. Among secondary endpoints, it improved multiple atherogenic lipid parameters, including apolipoprotein (Apo)-C3 and lipoprotein(a) (Lp(a)), small dense low-density lipoproteins, large very low-density lipoproteins, and chylomicrons. This reduction occurred after 6 months of treatment and was maintained up to 1 year [15]. Furthermore, a meta-analysis of 4 randomized, placebo-controlled trials confirmed that resmetirom (80 mg daily) significantly improved serum TGs, low-density lipoprotein cholesterol (LDL-C), Lp(a), and Apo-B levels [16]. Another meta-analysis confirmed these findings and found resmetirom to additionally lower Apo-C3 and increase adiponectin levels compared with placebo [17].

A model-based analysis estimated that resmetirom treatment may reduce 5-year and lifetime cardiovascular mortality, resulting in an overall gain of 0.45 life-years in patients with MASH and F2 liver fibrosis [18]. A matched cohort study that included 2380 adults with MASH found a significant association between its use and a reduced risk of MACE along with lower all-cause mortality [19]. To date, resmetirom remains one of the few MASH therapies with real-world evidence supporting cardiovascular benefit.

Resmetirom has demonstrated a favourable safety profile in clinical trials, with the most commonly reported adverse events being nausea and diarrhea, mostly mild or moderate in severity [20]. Pharmacovigilance data from the first year of real-world use identified gastrointestinal and hepatobiliary (including liver enzyme elevation, hyperbilirubinemia, and jaundice) adverse events as the most frequent, while cardiovascular events (chest pain, tachycardia, peripheral edema, and blood pressure abnormalities) were uncommon [21].

TERN-501 is a next-in-class THRβ full agonist that is being developed for the treatment of MASH as monotherapy or combined with TERN-101, a farnesoid X receptor (FXR) agonist. Recently, it has been evaluated in the multicenter, randomized, double-blind, placebo-controlled Phase 2a DUET trial. Treatment with TERN-501 at daily doses between 1 and 6 mg for 12 weeks resulted in dose-dependent, statistically significant reductions in liver fat content compared with placebo. At 6 mg, the treatment was also associated with a significant reduction in TGs, very low-density lipoprotein cholesterol (VLDL-C), and Lp(a). Both combination groups (3 or 6 mg TERN-501 plus 10 mg TERN-101) also demonstrated significant reductions from baseline in total cholesterol (TC) and Lp(a) compared with placebo [22].

Another full THRβ agonist, VK2809, has met its primary efficacy endpoint in VOYAGE, a 52-week-long, randomized, double-blind, placebo-controlled Phase 2b trial. Along with histological improvements in biopsy-confirmed MASH with fibrosis, VK2809 treatment (1–10 mg QD) resulted in significant reductions in TG, LDL-C, Lp(a), Apo-B, and Apo-C3 levels [23]. According to a press release, ASC41 also reduced TC, LDL-C, and TG levels by up to 23.4%, 27.7% and 46.5%, respectively, in the first 12 weeks of a year-long ongoing Phase 2 trial in subjects with biopsy-confirmed MASH (NCT05462353) [24,25].

Other THRβ agonists that are currently being evaluated in Phase 1 or 2 clinical trials include KYLO-0603, thybetaxor (CS060380), ALG-055009, CS060304, HSK31679, KH629, and HEC169584 (NCT07198386, NCT06342947, NCT06485245) [14,24,26]. Notably, several of those, specifically thybetaxor, VK2809, ASC41, HSK31679, ALG-055009, ECC-4703, and KH629, have different variants of dyslipidemia as alternative independent indications [14]. KYLO-0603 is reported to be the first THRβ agonist engineered utilizing N-acetylgalactosamine conjugation to enable targeted delivery to hepatocytes, reducing the risk of systemic adverse effects [26].

HPG7233 is currently entering clinical development as monotherapy for MASH and hyperlipidemia, and a combination comprising HPG7233 and the FXR agonist HPG1860 has advanced to Phase 2a trials in MASH [24]. ECC4703 has demonstrated meaningful synergism with the incretin mimetics semaglutide and tirzepatide in a murine model of diet-induced obesity [27], and is to be evaluated as a standalone option and in combination with ECC0509, a semicarbazide-sensitive amine oxidase (SSAO) inhibitor (NCT07288138) [24]. These data suggest that novel THRβ agonists may confer cardiovascular benefits in MASH, with or without fibrosis, and have strong potential to be used in combination regimens to enhance efficacy.

## 4. Incretin Mimetics

Incretins are gut-derived peptide hormones that regulate glucose homeostasis, energy expenditure, and satiety following food intake. There are two known incretins, the glucagon-like peptide-1 (GLP1) and glucose-dependent insulinotropic peptide (GIP), which are members of the glucagon peptide superfamily. GLP1 and GIP exert their effects by binding to specific receptors (GLP1Rs, GIPRs) that are widely expressed in the pancreas, gastrointestinal tract, cardiovascular system, and the brain. Incretins enhance insulin secretion, suppress glucagon release, inhibit hepatic gluconeogenesis, delay gastric emptying, and mediate satiety signaling in the central nervous system [28,29].

GLP1 and GIP may also act as a critical link connecting digestion, metabolism, and cardiovascular function, as they are known to improve endothelial function and blood pressure control, reduce cardiac workload and vascular resistance, optimize myocardial glucose use and support tissue perfusion to meet the body’s increased metabolic demands [28]. The possible cardioprotective mechanisms of incretin mimetics include reducing epicardial adipose tissue thickness, improving myocardial energy metabolism, inhibiting the renin–angiotensin–aldosterone system, reducing systemic inflammation and cardiac oxidative stress, and delaying the progression of atherosclerosis [30].

Glucagon is produced through the cleavage of the same propeptide (proglucagon) as the incretins and works in counterbalance with insulin to control glycemia and energy expenditure. Through activation of its receptors (GCGRs), glucagon stimulates glycogenolysis and suppresses glycogenesis as well as activates ketogenesis and mitochondrial fatty acid oxidation in the liver, and inhibits lipogenesis and promotes lipolysis in white adipose tissue. Additionally, it exerts chronotropic and inotropic effects, increasing cardiac output, contractility, and heart rate [31].

Historically, the first incretin mimetics to come into clinical use were selective GLP1 receptor agonists (GLP1RAs). However, based on preclinical and clinical data showing substantially increased therapeutic efficacy, recent development has largely focused on unimolecular dual (GLP1R/GIPR or GLP-1R/GCGR) and even triple (GLP1R/GIPR/GCGR) co-agonists [29]. Additionally, fixed combinations and unimolecular poly-agonists targeting incretin and amylin receptors (cagrilintide + semaglutide, petrelintide + CT-388, amycretin) are being explored for T2DM and obesity, and should be expected to eventually enter the MASLD pipeline as well [28,31].

### 4.1. GLP1R Agonists

Currently marketed GLP1RAs include semaglutide, liraglutide, dulaglutide, exenatide, lixisenatide, beinaglutide, ecnoglutide, and efsubaglutide alfa; two of them, semaglutide and liraglutide, are also approved for weight management while others are indicated for T2DM treatment [12]. In August 2025, semaglutide (Wegovy^®^ solution for subcutaneous injection, NN9535) became the first GLP-1RA to receive the US Food & Drug Administration’s (FDA) approval for the treatment of MASH with moderate-to-advanced (F2/F3) liver fibrosis in combination with dietary and lifestyle changes. Wegovy^®^ injection and tablets are also indicated for reducing MACE risk in adults with established CVD and either obesity or overweight [12]. A similar indication has been approved for the GLP1RAs liraglutide (Victoza^®^) and dulaglutide (Trulicity^®^), but their use is currently limited by T2DM populations [12].

Recent meta-analyses report that GLP-1RAs significantly improve MASH activity and liver histologic features, including hepatocellular ballooning, lobular inflammation, steatosis, and fibrosis [32,33,34]. Additionally, a network meta-analysis demonstrated that GLP1RAs were as effective as pioglitazone and vitamin E in terms of liver histology in MASH [35].

Over the last few years, at least 4 large-scale placebo-controlled trials were conducted in different patient populations receiving semaglutide [36]. The STEP-HFpEF program demonstrated that in obesity-related heart failure (HF) with preserved ejection fraction (HFpEF), semaglutide significantly improved symptoms, increased exercise capacity, reduced biomarkers of cardiac inflammation and congestion, and attenuated cardiac remodeling [37,38]. In the STEP-HFpEF DM trial enrolling diabetic subjects, it was superior to placebo in terms of ameliorating HF-related symptoms and physical limitations [39]. A pooled analysis of the two trials found a lower risk of worsening HF events with semaglutide compared with placebo [40]. In overweight/obese patients with atherosclerotic CVD who took part in the SELECT trial, semaglutide reduced the risk of MACE and composite HF endpoints compared with placebo. Statistically significant benefits were observed in those with and without clinical HF, regardless of HF subtype [41]. The FLOW trial investigators reported that semaglutide substantially reduced time to first HF event, time to cardiovascular death, and the composite of these outcomes [42].

Ultimately, a post hoc pooled analysis of all 4 trials found that semaglutide (2.4 mg once weekly (QW)) significantly reduced the risk of the combined endpoint of cardiovascular death or heart failure events, and worsening heart failure events, while not affecting cardiovascular mortality [36]. The cardiovascular effects of semaglutide directly correlated with weight loss and were more pronounced in participants with severe obesity [36], atrial fibrillation at baseline [43], and those receiving loop diuretics [44]. Furthermore, meta-analyses of randomized controlled trials revealed that semaglutide significantly improved lipid profiles [45] and lowered blood pressure in non-diabetic adults with overweight or obesity [46].

In a meta-analysis focused on its effects on safety and cardiovascular outcomes in overweight or obesity, semaglutide use was found to be associated with gastrointestinal adverse effects (nausea, vomiting, constipation, and diarrhea), with a subcutaneous route and higher doses (2.4 mg subcutaneously and 50 mg orally) presenting significantly higher risks [47]. In T2DM patients with high cardiovascular risk, semaglutide was not associated with increased frequency of severe or life-threatening conditions, such as severe hypoglycemia, retinopathy complications, acute pancreatitis, acute kidney failure, or malignancies [48]. Since GLP1RAs delay gastric emptying and may impact absorption of oral medications, their concomitant use with drugs that have a narrow therapeutic index may require intensified laboratory and/or clinical monitoring [12].

Liraglutide (1.8 mg QD) was safe, well-tolerated, and led to histological MASH resolution in the Phase 2 LEAN trial [49]; despite promising results, it is no longer being evaluated for this indication. Systematic reviews and meta-analyses of GLP1RA trials in people with T2DM showed reductions in the risk for MACE by 13–14%, myocardial infarction by 10–14%, stroke by 13–17%, cardiovascular death by 13–14%, all-cause death by 12%, and hospitalisation for heart failure by 11–14% [31]. Clinical evidence supports the inclusion of GLP1RAs in combination regimens to achieve better clinical outcomes of T2DM and/or MASLD; potential synergistic combinations may include sodium/glucose cotransporter 2 (SGLT2) inhibitors, FXR agonists, and/or lipogenesis inhibitors [50,51].

### 4.2. Dual GLP1R/GIPR Agonists

Preliminary data suggest that GLP1R/GIPR co-agonism not only provides enhanced glycemic control but can also favorably impact lipid profiles by reducing TGs and LDL-C, potentially offering cardiovascular protection [29]. Tirzepatide (Mounjaro^®^, Zepbound^®^, LY3298176) is the first-in-class, FDA-approved dual GLP1R/GIPR agonist (a so-called ‘twincretin’) indicated for T2DM, obesity/overweight, and obesity-related obstructive sleep apnea [12]. In a randomized, double-blind, Phase 2 trial, treatment with subcutaneous tirzepatide (5, 10, or 15 mg QW) for 52 weeks was more effective than placebo with respect to resolution of MASH without worsening of fibrosis [52]. The drug is to be evaluated in the SYNERGY-Outcomes Phase 3 study dedicated to the prevention of major adverse liver outcomes in people with high-risk MASLD (NCT07165028) [24].

Post hoc and meta-analyses report that tirzepatide is efficacious for reducing TGs, LDL-C, VLDL-C, Apo-C3, and Apo-B, and may be more effective in terms of ameliorating dyslipidemia than the selective GLP1RAs semaglutide and dulaglutide [53,54]. Among patients with T2DM and atherosclerotic CVD, tirzepatide significantly reduced the risk of death from cardiovascular causes, myocardial infarction, or stroke, and was non-inferior to the active comparator dulaglutide [55]. In a randomized, placebo-controlled trial enrolling obese subjects with HFpEF, tirzepatide therapy was associated with lower risks of death from cardiovascular causes or worsening HF, compared with placebo [56].

### 4.3. Dual GLP1R/GCGR Agonists

Dual GLP1R/GCGR agonists are sometimes referred to as oxyntomodulin analogs based on their functional similarity to oxyntomodulin, a peptide hormone produced by the oxyntic (fundic) cells of colonic mucosa. Whereas selective GCGR agonism would be undesired because of the hyperglycemic effects of glucagon, current investigational co-agonists are designed to have GLP1R:GCGR binding affinity ratios between 1:1 and 8:1 [29,57,58].

Pemvidutide (ALT-801) is an investigational peptide with balanced 1:1 GLP1R/GCGR receptor agonist activity. Two randomized, controlled, Phase 2 trials subjects demonstrated that treatment with pemvidutide (1.2, 1.8, or 2.4 mg QW) resulted in significant reductions in liver fat content and non-invasive inflammatory biomarkers in MASLD subjects [59], and promoted MASH resolution without worsening of fibrosis [60]. In these trials, all doses of pemvidutide produced significant improvements in systolic blood pressure [59,60], and 1.8 mg QW decreased serum TGs [60]. Furthermore, a lipidomic analysis of plasma samples from pemvidutide-treated patients revealed dose-dependent reductions in lipotoxic and pro-atherogenic lipid species [61].

Efinopegdutide (MK-6024) is a synthetic oxyntomodulin-derived peptide with a GLP1R:GCGR relative potency of approximately 2:1. In an open-label, active comparator-controlled, Phase 2a trial, efinopegdutide (10 mg QW) was superior to semaglutide in terms of reducing liver fat content and improving serum lipid profiles in MASLD patients [57]. A study in subjects with precirrhotic MASH has been completed with results pending (NCT05877547), and another one in participants with compensated cirrhosis due to MASH is ongoing (NCT06465186) [24]. Mazdutide (IBI362) is approved in China for weight management and T2DM, and is to be evaluated in Phase 2 trials in MASH subjects (NCT06937749) [24].

Survodutide (BI 456906), unlike other GLP1R/GCGR co-agonists, is a glucagon analog with sequence modifications introduced to balance receptor binding affinity with a slight preference for GCGR. In a 48-week, Phase 2 trial in patients with biopsy-confirmed MASH and F1 to F3 liver fibrosis, survodutide (2.4, 4.8, or 6.0 mg QW) reduced liver fat content by at least 30% in up to 67% of subjects and improved fibrosis by at least one stage in up to 36% subjects [62]. In a dose-finding Phase 2 trial in non-diabetic obese patients, significant improvements in blood pressure were observed with survodutide [63]. A randomized, double-blind, placebo-controlled, Phase 3 trial to evaluate its efficacy in cirrhosis due to MASH has started recruitment (NCT06632457) [24].

### 4.4. Triple GLP1R/GIPR/GCGR Agonists

Available animal and clinical data demonstrate that unimolecular GLP1R/GIPR/GCGR triagonists may have even greater efficacy in terms of weight loss than dual GLP1R/GIPR or GLP1R/GCGR agonists with apparently similar safety and tolerability profiles [29,31]. Retatrutide (LY3437943) has a higher affinity for the GIPR than endogenous GIP, but lower affinity for GLP1R and GCGR than the endogenous peptides, respectively [58]. In a 48-week Phase 2a trial in obese subjects without T2DM, retatrutide treatment (1–12 mg QW) was associated with significant improvements in body weight and multiple cardiometabolic measures, including systolic and diastolic blood pressure, glycated hemoglobin (HbA_1C_), fasting glucose, insulin, and serum lipids except HDL-C [64].

A substudy in participants who had visualization-confirmed MASLD revealed that retatrutide significantly decreased liver fat content, and this effect correlated with changes in body weight, abdominal fat and metabolic markers [65]. Trials have been initiated to assess retatrutide in obese individuals with concomitant CVD (NCT05882045), its potential impact on cardiovascular and kidney outcomes in obesity (NCT06383390), and on liver histology in non-cirrhotic MASH without T2DM (NCT04505436) [24].

Efocipegtrutide (HM15211) is a novel incretin triagonist with balanced receptor engagement and an extended half-life. In a Phase 1/2a trial in obese, non-diabetic patients with MASLD, it significantly decreased liver fat content and body weight across a dose range of 0.02–0.08 mg/kg over 8 or 12 weeks [66]. A randomized, double-blind, placebo-controlled, Phase 2 trial in biopsy-confirmed MASH is ongoing (NCT04505436) [24].

### 4.5. GPR119 Agonists

G protein-coupled receptor 119 (GPR119) is expressed in pancreatic β-cells and enteroendocrine cells, where its activation is required for insulin and GLP1 secretion, respectively. Additionally, GPR119 is found in hepatocytes, hepatic stellate cells, and Kupffer cells, where it is involved in the negative regulation of inflammation, lipid accumulation, and fibrogenesis [67]. Vanoglipel (DA-1241) is an orally available small-molecule GPR119 agonist, which has recently completed a 16-week, multicenter, randomized, double-blind, Phase 2a trial in subjects with presumed MASH. Alone (50 or 100 mg QD) or in combination with the dipeptidylpeptidase 4 inhibitor sitagliptin, vanoglipel significantly reduced liver steatosis, inflammation, and fibrosis, and improved glycemic control and plasma lipidomic profiles compared with placebo [68]. In mouse models, vanoglipel demonstrated significant synergism with semaglutide [69] and efruxifermin [70].

## 5. FXR Agonists

FXR, also known as the bile acid receptor, is a nuclear receptor expressed at high levels in the liver and intestine, where chenodeoxycholic and other bile acids act as its endogenous agonists. Hepatic FXR downregulates bile acid synthesis from cholesterol and suppresses the expression of sterol regulatory element-binding protein 1c, which is a critical positive regulator of key enzymes involved in de novo lipogenesis, including fatty acid synthase (FASN). FXR agonists were also shown to activate free fatty acid oxidation via peroxisome proliferator-activated receptor (PPAR) α-dependent pathways, decrease hepatic VLDL-C production, and promote TG hydrolysis [71,72].

Current pipeline includes at least 3 Phase 2 candidates, vonafexor, linafexor, and HPG1860, as well as several molecules in early development stages. In the LIVIFY study, vonafexor (EYP001), a non-steroidal FXR agonist, provided significant reductions in liver fat content, liver enzyme levels, serum lipids, and body weight versus placebo after 12 weeks [73]. Patients treated with vonafexor also showed improvements in glomerular filtration rate, which led to its subsequent repurposement for mild or moderate renal impairment with suspected MASH (NCT06939816) [24].

In the double-blind, placebo-controlled, dose-ranging Phase 2a RISE trial, HPG1860 (3–8 mg QD for 12 weeks) significantly reduced liver fat content and serum transaminases, but did not affect LDL-C levels [74]. A Phase 2, randomized, double-blind, placebo-controlled study of linafexor (CS0159) in MASH patients (NCT05591079) has been completed, but published results are not available yet [24]. A meta-analysis of 8 randomized trials confirmed that treatment with different FXR agonists significantly reduced liver steatosis, improved serum levels of liver transaminases and γ-glutamyltransferase, but had no significant effect on alkaline phosphatase. It must be noted that the analysis included several trials of FXR agonists not anymore under active development for MASLD/MASH, namely obeticholic acid, cilofexor (GS-9674), tropifexor (LJN452), and EDP-305 [75].

So far, clinical evidence supporting cardiovascular benefits of FXR agonists appears to be lacking, although animal studies have demonstrated potentially relevant effects on cardiac and cardiometabolic parameters. GW4064, a potent, non-steroidal FXR agonist, attenuated post-myocardial infarction cardiac remodelling and dysfunction in mice, possibly via an adiponectin-dependent mechanism [76]. In leptin-resistant diabetic mice, it ameliorated aortic medial hypertrophy, improved cardiomyocyte disarray and left ventricular mass index, along with a beneficial impact on systemic inflammation and lipid metabolism [77]. A novel candidate molecule for noncirrhotic MASH, designated HEC96719, has been developed based on the structure of GW4064, with modifications introduced to optimize tissue specificity and potency [78].

Obeticholic acid, a FXR agonist used to treat primary biliary cholangitis, effectively attenuated pulmonary arterial wall thickening, reduced right ventricular hypertrophy, and improved exercise capacity in a rat model of pulmonary hypertension [79]. GW4064, obeticholic acid, and another selective FXR agonist, PX20606, all demonstrated differential effects on cholesterol and lipoprotein parameters in mice and cynomolgus monkeys. Notably, PX20606 was able to produce a pronounced decrease in atherosclerotic plaque size in mice with genetic dyslipidemia [80]. To the best of our knowledge, none of these molecules is currently under active development for the treatment of MASLD/MASH. However, available preclinical data appear to support the rationale for combining FXR and THRβ agonists, as illustrated by the previously mentioned TERN-501/TERN-101 combination [22].

## 6. PPAR Agonists

PPARs, including PPARα, PPARγ, and PPARβ/δ, are a family of ligand-activated transcription factors that play essential roles in regulating carbohydrate and lipid metabolism, cell cycle progression, differentiation, and proliferation. PPARα, activated by fibrates, is highly expressed in the liver, where it regulates fatty acid metabolism, including β-oxidation and ketogenesis [81]. PPARγ, the molecular target of thiazolidinediones, improves insulin sensitivity, promotes lipid uptake and utilization, and regulates glucose metabolism in adipocytes, while also enhancing adiponectin secretion and upregulating fibroblast growth factor (FGF) 21 [81].

PPARβ/δ, for which no selective agonists are currently approved for clinical use, is involved in the regulation of fatty acid catabolism in skeletal muscle as well as in the maintenance of insulin sensitivity and systemic lipid homeostasis [81]. While selective PPARα and -γ agonists are already used worldwide to treat dyslipidemia and T2DM, respectively, the MASLD/MASH development pipeline includes a number of novel dual- and even pan-PPAR-activating candidates.

### 6.1. PPARα Modulators

Pemafibrate (Parmodia^®^, K-877), a selective PPARα modulator (SPPARMα), is marketed in Japan for the treatment of atherosclerotic disease. Within the emergent SPPARMα framework, this agent is regarded as a member of a novel therapeutic class, distinct from fibrates due to its high tissue and effect specificity as well as a favorable hepatic safety profile [82]. Pemafibrate improved liver enzymes and non-invasive fibrosis markers in a single-arm, prospective study enrolling subjects with MASLD and dyslipidemia [83]. In patients with T2DM and mild-to-moderate hypertriglyceridemia, it lowered TG, VLDL-C, remnant cholesterol, and Apo-C3 levels, but did not affect the incidence of cardiovascular events or associated mortality [84]. Improvements in cardiac remodeling and fibrosis were observed with pemafibrate treatment in preclinical pressure-overload heart failure models [85], and one small-sized observation study found that pemafibrate restored left ventricular diastolic function in diabetic patients with hypertriglyceridemia [86].

Currently, standalone pemafibrate is being investigated for MASLD complicated by hypertriglyceridemia (NCT06623539), primary biliary cholangitis (PBC) and dyslipidemia [24]. In the MASH indication, it is being evaluated as part of a combination (K-001) with tofogliflozin (Apleway^®^, Deberza^®^, CSG452), an SGLT2 inhibitor approved in Japan to treat T2DM. Similar to several other SGLT2 inhibitors, tofogliflozin has previously demonstrated to improve liver function and cardiometabolic markers in patients with T2DM, obesity and/or MASLD [87,88]. Since pemafibrate monotherapy was associated with a higher incidence of adverse renal events and venous thromboembolism [84], it appears that combining it with other liver-targeting agents may be necessary to maintain a favorable benefit-risk profile.

### 6.2. Dual Agonists

Dual PPARα/γ agonists ameliorate hypertriglyceridemia and mixed dyslipidemia through PPARα activation, and improve glycemic control via PPARγ-dependent pathways. Saroglitazar (saroglitazar magnesium, ZY-H1), the first in its class, is currently marketed in India as Lipaglyn^®^ for the treatment of T2DM and diabetic dyslipidemia, and as Bilypsa^®^ for MASLD/MASH [89]. In a randomized, placebo-controlled, double-blind, Phase 2 study in MASH subjects, saroglitazar (4 mg QD for 16 weeks) improved liver steatosis and atherogenic dyslipidemia, with notable reductions in adiponectin, TGs, and the Homeostatic Model Assessment for Insulin Resistance (HOMA-IR) index [90].

A meta-analysis of 7 randomized clinical trials established that saroglitazar significantly lowered alanine aminotransferase, TG and LDL-C levels versus placebo, albeit with certain safety concerns, specifically an increase in serum creatine [91]. Post-marketing programs are ongoing to assess saroglitazar’s effectiveness for the treatment of MASH with comorbidities (either obesity, T2DM, dyslipidemia, or metabolic syndrome) (NCT05872269), while Phase 3 trials are planned for a new indication, PBC (NCT07216235) [24]. Another PPARα/γ agonist, aleglitazar (no longer under active development), did not reduce the risk of cardiovascular outcomes in T2DM patients and a recent acute coronary syndrome [92], which highlights the need for careful evaluation of the potential cardiovascular benefits of glitazars in future studies.

Elafibranor (GFT505), a dual PPARα/δ agonist, was studied extensively in MASH but discontinued for this indication after showing inadequate efficacy in the Phase 3 RESOLVE-IT trial [93]. In late 2024, elafibranor was approved by the FDA as Iqirvo^®^ tablets for the treatment of PBC in adults unable to take ursodeoxycholic acid (UDCA) or with suboptimal response to UDCA monotherapy [94].

A study in Syrian hamsters with diet-induced MASH accompanied by heart failure found that elafibranor resolved liver injury, with significant reduction in ballooning and fibrosis scores, and improved diastolic dysfunction [95]. Additionally, elafibranor ameliorated hypercholesterolemia and severe hypertriglyceridemia in a mouse model of metabolic syndrome [96]. ZSP0678, another dual PPARα/δ agonist, has recently completed Phase 1 trials (NCT04137055) [16].

### 6.3. Triple PPAR Agonists

Lanifibranor (IVA337) is an orally available small molecule acting as a pan-PPAR agonist with balanced α-, γ- and β/δ-subtype activity [97]. In the randomized Phase 2b study NATIVE that enrolled MASH patients, 76% of whom had moderate or advanced fibrosis, lanifibranor (800 or 1200 mg QD for 24 weeks) was superior to placebo in terms of MASH resolution, improvement in fibrosis stage, and the composite of both outcomes [98]. A post hoc analysis found that TG, HDL-C, apolipoproteins, insulin, HOMA-IR, HbA_1C_, fasting glucose, C-reactive protein, ferritin, and diastolic blood pressure were improved significantly by lanifibranor. Notably, these beneficial effects correlated with increases in adiponectin levels, but were observed regardless of diabetes status and weight change [97]. Moreover, 35% to 44% of patients at high baseline cardiovascular risk shifted to intermediate or low risk after 24 weeks of lanifibranor treatment across different dose arms, compared with 13% to 26% in the placebo arm [97].

In a Syrian hamster model of comorbid MASH, obesity, and heart failure with preserved ejection fraction, lanifibranor improved cardiac function, TGs, fasting plasma glucose, insulin, and HOMA-IR, while also ameliorating hepatic steatosis, inflammation, and fibrosis. Notably, lanifibranor showed equal cardiometabolic benefits to the GLP1R agonist semaglutide, but had superior effects in the liver [99]. This compound has also been reported to attenuate right ventricular hypertrophy in a mouse model of systemic sclerosis and pulmonary hypertension [100], and reduce portal hypertension in mice by improving vascular and hepatic sinusoidal function independent of liver fibrosis, possibly through its anti-angiogenic effects [101].

Chiglitazar (carfloglitazar, CS-038) is a pan-PPAR-agonist approved in China as Bilessglu^®^ tablets for the treatment of T2DM. A randomized Phase 2 study in T2DM patients showed that chiglitazar treatment (32 or 48 mg QD over 16 weeks) was associated with a marked reduction in serum TGs along with improvements in glycemic control [102]. When used as an add-on to metformin in subjects with inadequately controlled T2DM, it achieved meaningful reductions in TG and free fatty acid levels, and significantly increased serum HDL-C [103]. Phase 2 trials are ongoing to evaluate chiglitazar’s effectiveness for MASH cirrhosis (NCT06773221) and MASLD in T2DM patients (NCT07303803) [24].

Free fatty acid receptor (FFAR) 1 is a transmembrane receptor involved in the regulation of insulin secretion, hepatic insulin resistance, lipid accumulation, and fibrogenesis. RLA8 and ZLY18, experimental quadruple FFAR1/PPARα/γ/δ agonists, have been reported to exert potent antisteatotic, anti-inflammatory, and antifibrotic effects in vivo [104,105]. Furthermore, recent advances in unimolecular polypharmacology have resulted in the development of a quintuple GLP1R/GIPR/pan-PPAR agonist. The compound was significantly more effective than either GLP1R/GIPR or pan-PPAR agonists alone, or when administered as a loose combination, in reducing body weight and enhancing glucose control in obese diabetic mice [106].

## 7. Lipogenesis Inhibitors

Inhibition of hepatic de novo lipogenesis and TG synthesis represents a therapeutic strategy aimed at reducing intrahepatic lipid accumulation and potentially attenuating lipotoxicity-driven inflammation and fibrogenesis. Within this approach, the principal enzymatic targets include acetyl-coenzyme A (CoA) carboxylase (ACC), FASN, stearoyl-CoA desaturase 1 (SCD1), and acyl-CoA:diacylglycerol acyltransferase (DGAT), all of which are required for the rate-limiting steps of fatty acid and TG synthesis [107,108]. Another viable approach is based on inhibition of the mitochondrial pyruvate carrier (MPC), a gating transport link in the major hepatic lipogenic acetyl-CoA production pathway [109].

### 7.1. Enzyme Inhibitors

Denifanstat (TVB-2640), an orally administered, selective FASN inhibitor, was evaluated in the randomized, double-blind, placebo-controlled, Phase 2b FASCINATE-2 trial in patients with biopsy-confirmed MASH and moderate-to-advanced fibrosis [110]. As monotherapy (50 mg QD over 52 weeks), it produced significant improvements in disease activity and MASH resolution without a worsening of fibrosis. LDL-C cholesterol was significantly reduced from baseline, and the ratio of polyunsaturated to saturated TGs increased by 107% in the intervention arm [110]. Recently, denifanstat has also completed a drug–drug interaction study with the THRβ agonist resmetirom, with plans to develop a combined regimen (NCT07216313) [24].

Ervogastat (PF-06865571), an oral, selective DGAT2 inhibitor, was discontinued as a standalone therapy and as part of a combination with the ACC inhibitor clesacostat (PF-05221304) [111], but is also to be evaluated for potential synergism with resmetirom [112]. Notably, the ervogastat/clesacostat combination was associated with a likely undesirable fasting lipid and apolipoprotein profile in MASH patients [111]. Another ACC inhibitor, firsocostat (GS-0976), has been evaluated in combination with the FXR agonist cilofexor, and/or the GLP1RA semaglutide to enhance therapeutic efficacy [113]. Thus, incorporating lipogenesis inhibitors into combination regimens may represent a strategy worth exploring for MASLD accompanied by atherogenic dyslipidemia.

ION224 (AZD2693) is an antisense oligonucleotide targeting DGAT2 messenger ribonucleic acid (mRNA). In the multicenter, randomized, double-blind, Phase 2 ION224-CS2 trial enrolling subjects with biopsy-confirmed MASH, F1-F3 fibrosis, and liver steatosis ≥ 10%, ION224 significantly reduced MASH activity without worsening of fibrosis compared with placebo [114]. Despite the apparent halt in the clinical development of ION224, these results support further exploration of antisense-mediated inhibition of de novo lipogenesis in MASLD.

Aramchol (arachidylamidocholanoic acid, icomidocholic acid, C20-FABAC) is a synthetic fatty acid-bile acid conjugate acting as an SCD1 inhibitor. In a Phase 2b trial, aramchol significantly reduced HbA_1C_ levels when compared to placebo, but had no appreciable effects on serum lipids [115]. In the non-randomized, open-label ARCON cohort study, aramchol treatment (300 mg twice daily (BID) for 24–72 weeks) was associated with a significant improvement in MASH fibrosis [116]. Based on these interim results, the randomized, double-blind, placebo-controlled Phase 3 ARMOR trial has been suspended, and further development is expected to be focused on aramchol meglumine instead of aramchol free acid (NCT06502561, NCT07251712) [24].

### 7.2. MPC Inhibitors

The MPC, also known as the mitochondrial target of thiazolidinediones, is an inner mitochondrial membrane protein that mediates the rate of pyruvate entry into the mitochondria. Inhibiting MPC can shift mitochondrial metabolism from glucose to using fatty acids and amino acids, promote mitochondrial biogenesis and function, increase insulin sensitivity, and inhibit de novo lipogenesis. Given their mechanism of action, MPC-inhibiting agents are being developed for MASLD/MASH in patients with concomitant obesity, insulin resistance and/or T2DM, and are expected to form synergistic combinations with incretin mimetics [109,117].

Azemiglitazone (MSDC-0602K) is a novel insulin sensitizer structurally similar to thiazolidinediones but designed to preferentially target the MPC. In the randomized, double-blind, placebo-controlled, Phase 2b EMMINENCE study, treatment with azemiglitazone (62.5, 125, or 250 mg QD for 52 weeks) was associated with significant reductions in liver enzyme levels and MASH activity with additional improvements in markers of glucose metabolism [118]. A post hoc analysis revealed that the addition of any dose of azemiglitazone to patients receiving GLP1R agonists for concomitant T2DM further improved fasting glucose, HbA_1C_, insulin, and liver enzymes [119].

Racemic pioglitazone, a non-selective PPARγ agonist and MPC inhibitor, is typically used as an antidiabetic agent and has some evident hepatic and metabolic benefits in MASLD. However, its clinical utility is limited by PPARγ-driven side effects, particularly water retention, which may precipitate heart failure decompensation, weight gain, and risk of fractures [4,120]. Given these considerations, a novel deuterium-stabilized R-pioglitazone enantiomer, PXL065, was designed to preferentially target the MPC while minimizing PPARγ binding [121].

In the randomized, Phase 2 DESTINY-1 study, PXL065 (7.5–22.5 mg QD for 36 weeks) significantly reduced liver fat content, HbA_1C_, and insulin resistance markers, and increased serum adiponectin versus placebo [121]. 10-week treatment with PXL065 significantly ameliorated left ventricular hypertrophy and cardiac fibrosis in a genetic mouse model of hypertrophic cardiomyopathy [122]. Despite the absence of definitive clinical evidence, available data suggest that MPC inhibitors and GLP1RAs have complementary mechanisms of action and can be combined to augment their therapeutic efficacy.

## 8. FGF21 Analogs

FGF21 is a peptide hormone secreted primarily by the liver (a so-called hepatokine), which enhances insulin sensitivity, stimulates glucose uptake by the adipose tissue and fatty acid oxidation, suppresses de novo lipogenesis, increases energy expenditure, and modulates nutrient preference via central mechanisms [123,124]. Elevated circulating FGF21 levels, possibly reflecting tissue FGF21 resistance, were associated with a higher risk of coronary heart disease events in patients using statins [125] and carotid atherosclerosis, independent of other risk factors [126]. In rodent models, exogenous FGF21 and its analogs have been found to improve cardiomyocyte survival and preserve ventricular function following myocardial infarction [127], reduce atherosclerotic lipid deposition and plaque area [128], attenuate diabetic cardiomyopathy [129], and rescue cardiac function in various pressure-overload, ischemia–reperfusion, and cardiotoxicity models [130].

Pegbelfermin (BMS-986036), one of the first FGF21 analogs to enter clinical trials, was safe and reduced liver fibrosis in the randomized, placebo-controlled, Phase 2 FALCON-1/2 studies, but failed to meet the primary endpoints and was discontinued [131,132]. Subsequent development has focused on next-generation FGF21 analogs with longer half-lives [123], whereas attempts to directly target the FGF21 receptor using agonistic antibodies (fazpilodemab (BFKB8488A) and MK-3655) were halted, possibly due to concerns regarding their benefit-risk profiles [133,134].

As of now, there are at least 3 drug candidates of the FGF21 analog class undergoing clinical trials, namely, pegozafermin (BIO89-100), efruxifermin (AKR-001), and efimosfermin alfa (BOS-580). Pegozafermin is a novel glycopegylated, long-acting FGF21 analog that is being evaluated in Phase 3 trials as a potential therapy for fibrotic and cirrhotic MASH as well as severe hypertriglyceridemia. In the Phase 2b ENLIVEN trial enrolling MASH patients with biopsy-proven F2/F3 fibrosis, pegozafermin treatment (30 mg QW or 44 mg every 2 weeks) led to statistically significant reductions in liver fat content, biomarkers of fibrosis and liver injury, along with improvements in serum lipid profiles [135].

Efruxifermin is a fusion protein with a half-life of approximately 3 days, developed for once-weekly subcutaneous injection. As reported by a systematic review with meta-analysis, efruxifermin (28 or 50 mg QW for 12 to 96 weeks) was superior to placebo in terms of MASH resolution and improvement in fibrosis as well as reductions in hepatic fat fraction and marker enzyme levels in 4 randomized trials. Moreover, patients receiving the drug achieved significant improvements in insulin sensitivity markers, serum adiponectin and HDL-C, and reductions in TG, LDL-C, Apo-B and Apo-C3 levels [136].

Efimosfermin alfa is an immunoglobulin G1-Fc-FGF21 fusion protein that has an even longer half-life (approximately 21 days), which makes it suitable for once-monthly administration. In a randomized, placebo-controlled, Phase 2a trial, efimosfermin alfa (75 or 150 mg once or twice a month, or 300 mg once-monthly) caused a significant reduction in hepatic fat fraction in patients with phenotypic MASH [137]. Studies are underway to evaluate its effectiveness in biopsy-confirmed MASH with F2/F3 fibrosis (NCT07221227) and cirrhosis (NCT06920043) [24]. In addition to histological improvements, treatment with efimosfermin alfa was associated with an 79% increase in circulating adiponectin and a slight decrease in TG levels [138].

According to a meta-analysis, pegozafermin (30 mg) and efruxifermin (28 or 50 mg) treatment was associated with significant improvements in non-HDL-C concentrations, while efimosfermin alfa (75 or 150 mg) and efruxifermin (50 mg) increased serum adiponectin levels [139]. Despite somewhat promising data from animal studies, definitive clinical evidence of any cardiovascular benefits of pharmacological FGF21 enhancement appears to be lacking.

## 9. PNPLA3 Antagonists

Patatin-like phospholipase domain-containing protein 3 (PNPLA3), also known as adiponutrin, is an enzyme that catalyzes triacylglycerol hydrolysis in hepatocytes and stellate cells. Adequate PNPLA3 activity is necessary to avoid hepatic retention of polyunsaturated fatty acids (PUFAs) and TGs, impairment of lipoprotein turnover, and mitochondrial dysfunction. The *PNPLA3*(148M) variant has a missense mutation that reduces PNPLA3 hydrolase activity and interferes with adipose triglyceride lipase-mediated TG hydrolysis, thereby promoting hepatic steatosis [140]. Recent studies have identified the *PNPLA3*(148M) polymorphism as a major causative factor in the development of liver steatosis, inflammation and fibrosis, potentially being the strongest inheritable genetic determinant of MASLD/MASH [141].

There is emerging evidence suggesting that PNPLA3 activity may also be linked to cardiovascular risk, which has made it an appealing therapeutic target for MASH with cardiometabolic comorbidities. A Mendelian randomization study found that genetically predicted PNPLA3 impairment significantly increased the risk of coronary atherosclerosis, coronary heart disease, and myocardial infarction, with suggestive associations observed for risk of heart failure and atrial fibrillation [142].

So far, PNPLA3-targeted therapies are still in preclinical or early clinical development, and there is no substantial evidence on whether they could provide any cardiometabolic benefits for MASH patients carrying the (148M) mutation. AZD2693, an antisense oligonucleotide targeting hepatic PNPLA3 mRNA, dose-dependently reduced liver fat content and increased PUFAs in serum TGs in Phase 1 studies in noncirrhotic MASH [143], but was discontinued from Phase 2 after demonstrating insufficient efficacy [144].

ARO-PNPLA3 (JNJ-75220795), a hepatocyte-specific small interfering RNA (siRNA), induced an up to 46% reduction in liver fat content 12 weeks following the administration of a single dose. The effect was restricted to homozygous carriers of the (148M) variant, which reinforced the concept that anti-PNPLA3 therapies may represent a precision-medicine approach for individuals with a genetic predisposition to MASLD [145]. LY3849891 is another anti-PNPLA3 siRNA that has recently entered a Phase 1 trial in (148M)-carrying MASLD patients (NCT05395481) [24].

## 10. Galectin Antagonists

Galectin-3 is a β-galactoside-binding lectin with widespread tissue expression and a broad functionality, including regulation of cell growth and differentiation, cell adhesion, cell cycle, apoptosis, and antitumor response. Galectin-3 promotes transforming growth factor β1 (TGFβ1) signaling, myofibroblast activation and proliferation, collagen expression and cross-linking, thereby acting as a key positive regulator of fibrogenesis [146,147]. In animal models of heart failure, galectin-3 has been found to promote inflammation and fibrogenesis, leading to accelerated cardiac remodeling and dysfunction [148]. Additionally, observational studies reported significant associations between higher plasma galectin-3 levels and increased risk of ischemic stroke, coronary heart disease, heart failure, cardiovascular and all-cause mortality [149,150].

Investigational galectin-3 inhibitors are being developed to treat MASH with advanced liver fibrosis, cirrhosis, portal hypertension, idiopathic pulmonary fibrosis, and several kinds of solid tumors [151,152]. In a Phase 2b trial including patients with cirrhotic MASH, 1 year of belapectin (GR-MD-02) at 2 mg/kg did not achieve a significant reduction in portal hypertension or liver fibrosis except in a subgroup of participants without esophageal varices [151]. In NAVIGATE, a Phase 2b/3 trial, belapectin significantly reduced liver stiffness measurement in patients with MASH cirrhosis, confirming its potent antifibrotic activity [153]. Another galectin-3 inhibitor, selvigaltin (GB1211), has demonstrated safety in participants with hepatic impairment in the Phase 1b/2a GULLIVER-2 program [154]

Several experimental anti-galectin agents, including citrus pectin, N-acetyl-lactosamine, and G3-C12 have been demonstrated to offer protection against myocardial infarction and ischemia/reperfusion injury [155,156] as well as reduce atherosclerotic plaque burden in animal studies [157]. Despite apparent mechanistic links, it seems that no clinical studies have yet directly addressed the cardiovascular impact of galectin-3 inhibition.

## 11. Miscellaneous

Berberine ursodeoxycholate (BUDCA, HTD1801) combines the structural parts and biological effects of two liver-targeting molecules with anti-inflammatory and anticholestatic properties: UDCA and the isoquinoline alkaloid berberine. These respective moieties contribute to the key mechanisms of BUDCA’s action, specifically activation of adenosine monophosphate kinase and NLR family pyrin domain-containing 3 (NLRP3) inflammasome inhibition [158,159]. Given that both berberine [160] and UDCA [161,162] have demonstrated beneficial cardiometabolic effects in animal studies, BUDCA appears to be a promising drug candidate for the treatment of MASH concomitant with T2DM, dyslipidemia, and atherosclerotic disease.

Placebo-controlled Phase 2 studies found that BUDCA treatment was associated with improvements in liver steatosis, transaminase levels, and glycemic control in patients with T2DM and suspected MASH [163]. In the randomized, double-blind, active comparator-controlled, Phase 3 HARMONY trial, BUDCA (500 or 1000 mg BID) achieved a greater reduction in HbA_1C_ at week 24 than dapagliflozin in adults with T2DM inadequately controlled with metformin. It also demonstrated superior improvements in cardiometabolic markers, including Lp(a), LDL-C and non-HDL-C levels, and rate of statin therapy intensifications [159].

In subjects with a history of hypercholesterolemia and elevated serum LDL-C levels, the highest dose of BUDCA (2000 mg QD) provided significant reductions in serum TC and LDL-C, but no changes in TG and HDL-C levels compared with placebo [164]. Additionally, results of a multicenter observational program demonstrated that augmenting statin therapy with UDCA increased its efficacy in chronic liver disease patients with high cardiovascular risk [165].

Namodenoson (CF102) is an orally available synthetic purine nucleotide that selectively activates the A3 adenosine receptor (A3AR) and exerts anti-inflammatory and antitumor effects via phosphoinositide 3-kinase-dependent pathways [166]. In a randomized, placebo-controlled Phase 2a trial, namodenoson treatment (12.5 or 25 mg BID for 12 weeks) was associated with dose-dependent reductions in serum transaminase levels in MASH subjects [167]. A Phase 2b trial is ongoing to evaluate namodenoson’s safety and effectiveness in up to 114 adults with biopsy-confirmed MASH and F1-F3 liver fibrosis (NCT04697810) [24].

The cardioprotective effects of namodenoson are believed to be mediated by an upregulation of adiponectin signaling pathways in an A3AR-dependent fashion. In murine models of ischemia–reperfusion injury, namodenoson reduced the size of myocardial infarct, attenuated left ventricular dysfunction, and limited leukocyte infiltration into the myocardium. Additionally, namodenoson administration ameliorated doxorubicin-induced oxidative stress, mitochondrial damage, and cardiomyocyte apoptosis, leading to cardiac dysfunction [168].

Icosabutate (NST-4016) is a semi-synthetic long-chain fatty acid that activates hepatic FFAR1 and -4 (also known as GPR40 and GPR120, respectively), and is more potent and selective than naturally occurring ω3-PUFAs, particularly docosahexaenoic and eicosapentaenoic acids [169]. A systematic review and meta-analysis confirmed that ω3-PUFA treatment is significantly associated with reductions in MACE risks. However, ω3-PUFAs increased incidental atrial fibrillation, and eicosapentaenoic acid monotherapy was associated with a higher risk of total bleeding events. Overall, the data on ω3-PUFA’s efficacy and safety in cardiovascular disease remain controversial [170,171,172].

Dual FFAR1/4 activation by icosabutate improves glycemic control and ameliorates hepatic inflammation and fibrosis via the β-arrestin 2 pathway, blocking both TGFβ-activated kinase 1 and the NLRP3 inflammasome. In the multicenter Phase 2b ICONA trial in patients with MASH and F1-F3 fibrosis, icosabutate (300 or 600 mg QD for 52 weeks) did not meet its primary efficacy endpoint (MASH resolution with no worsening of fibrosis), but improved liver enzymes and several metabolic biomarkers [169].

In a randomized, placebo-controlled trial in statin-treated patients with residual hypertriglyceridemia, icosabutate (600 mg QD for 12 weeks) significantly reduced TG, VLDL-C, non-HDL-C, and Apo-C3 levels, and improved HDL-C while having no effect on LDL-C [173]. Significant benefits of icosabutate treatment were also observed in a randomized, double-blind, placebo-controlled trial in patients with severe hypertriglyceridemia [174]. Additionally, icosabutate may reduce hepatic oxidative stress and the formation of oxylipins (oxidized PUFAs), including oxidized phospholipids [175], which are sometimes regarded as novel circulating markers of CVD [176].

ECC0509 is a small-molecule selective inhibitor of SSAO, also known as vascular adhesion protein 1 and primary amine oxidase. SSAO is highly expressed in the vascular system, especially on the surface of endothelial cells, where it regulates the adhesion and infiltration of circulating leukocytes, and in adipocytes, where it facilitates glucose uptake through the GLUT4 transporter. Elevated circulating soluble SSAO levels are evident in patients with all kinds of CVD including coronary artery disease, arterial stiffness, aortic stenosis, chronic HF, hypertension, and stroke [177,178].

In Apo-E-deficient mice and cholesterol-fed rabbits, the SSAO inhibitor PXS-4728A reduced atheroma formation, oxidative stress, and endothelial dysfunction, suppressed the expression of adhesion molecules and inflammatory cytokines, inhibited macrophage recruitment and activation, and decreased vascular smooth muscle proliferation [179,180]. PXS-4728A met the pre-specified endpoints in a Phase 2 study in MASH, but was discontinued due to a potentially harmful interaction with an undisclosed drug stated to be commonly used by MASH patients [181]. ECC0509 is being evaluated in a Phase 2 trial alone and in combination with the THRβ agonist ECC4703 in adults with presumed MASH (NCT07288138) [24].

DT-109 (diapin) is a glycine-glycine-L-leucine tripeptide that was selected as a drug candidate based on preclinical studies showing enhanced GLP1 and insulin secretion, and decreased blood glucose in both healthy and diabetic mice [182]. In a murine model of diet-induced MASH, DT-109 stimulated fatty acid oxidation, ameliorated hepatic inflammation and fibrosis by downregulating nuclear factor κB and TGFβ pathways, and improved body composition and serum lipid profiles [183]. DT-109 treatment may reduce atherosclerotic plaque burden and attenuate coronary artery disease via induction of glutathione biosynthesis independently of lipid-lowering effects, as demonstrated by a study in ApoE-knockout mice [184].

Leronlimab is a monoclonal antibody targeting the CC-motif chemokine receptor (CCR)-5 that is being developed for MASH, liver fibrosis, and a number of malignancy types [185]. To the best of our knowledge, there is no direct evidence of leronlimab’s cardiovascular effects; however, another anti-CCR5 agent, the antiretroviral maraviroc, has been found to significantly improve endothelial function, arterial stiffness, and early carotid atherosclerosis in HIV-suppressed patients at high cardiovascular risk [185]. Cenicriviroc (TAK-652), a dual CCR2/CCR5 antagonist, also demonstrated reductions in markers associated with cardiovascular outcomes, including high-sensitivity C-reactive protein and fibrinogen, in MASH patients [186], but was discontinued due to inadequate efficacy [187].

TB-840 (JC1-40) is an orally available thiourea derivative that acts as a retinoid-related orphan receptor α (RORα) agonist and has recently completed first-in-human trials [188]. While there is no direct evidence of TB-840’s cardiovascular effects, numerous in vivo studies suggest that RORα activation may enhance atherosclerotic plaque stability and ameliorate vascular endothelial dysfunction, ischemia-induced angiogenesis, myocardial ischemia–reperfusion injury, pathological myocardial hypertrophy, and diabetic cardiomyopathy [189].

**Figure 1:**
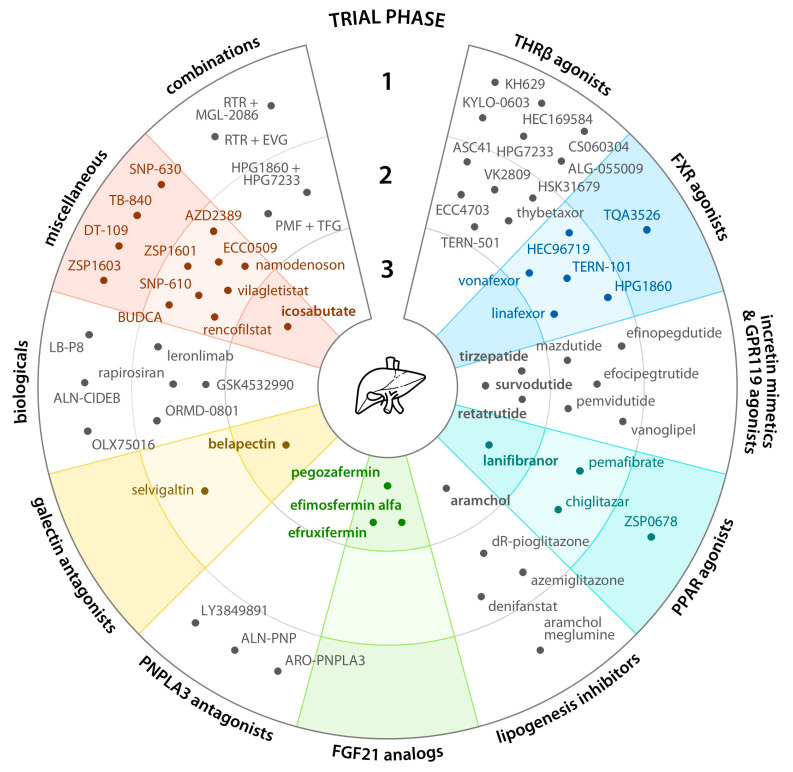
Drug candidates currently under clinical development for MASLD. BUDCA, berberine ursodeoxycholate; EVG, ervogastat; FGF21, fibroblast growth factor 21; FXR, farnesoid X receptor; GPR119, G protein-coupled receptor 119; PMF, pemafibrate; PNPLA3, patatin-like phospholipase domain-containing protein 3; PPAR, peroxisome proliferator–activated receptor; RTR, resmetirom; TFG, tofogliflozin; THRβ, thyroid hormone receptor β.

**Figure 2:**
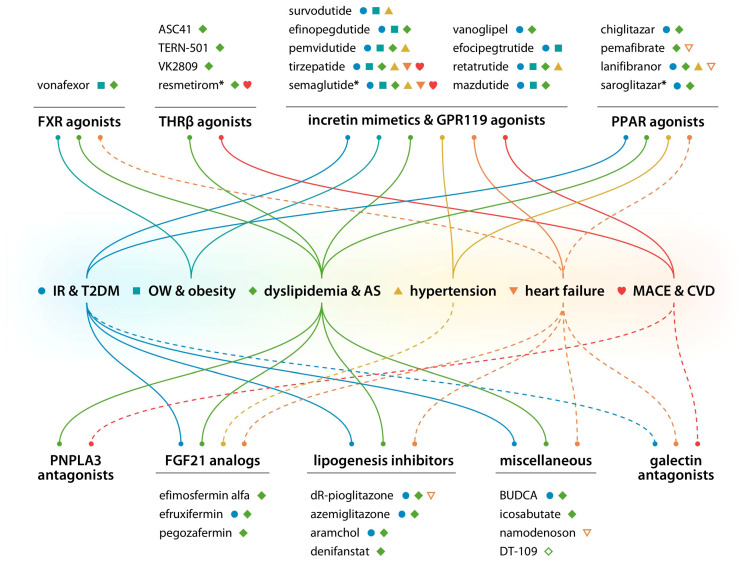
Cardiovascular effects of individual drug candidates, their classes, and approved first-in-class agents (marked with asterisks), based on clinical trial/real-world evidence (solid lines, filled pictograms) or on preclinical/mechanistic data (dashed lines, outlined pictograms). AS, atherosclerosis; BUDCA, berberine ursodeoxycholate; CVD, cardiovascular death (outside of MACE); FGF21, fibroblast growth factor 21; FXR, farnesoid X receptor; GPR119, G protein-coupled receptor 119; IR, insulin resistance; MACE, major adverse cardiovascular events; OW, overweight; PNPLA3, patatin-like phospholipase domain-containing protein 3; PPAR, peroxisome proliferator–activated receptor; T2DM, type 2 diabetes mellitus; THRβ, thyroid hormone receptor β.

The current clinical development pipeline for MASLD is presented in Figure 1, and the cardiovascular effects of individual drug candidates and their classes are summarized in Figure 2. Comparative characteristics of the main drug classes and their evidence base are presented in Table 1.

## 12. Conclusions and Future Perspectives

Despite the richness and diversity of the MASLD drug development landscape, the evidence regarding the potential cardiovascular benefits of most candidate molecules remains limited. Real-world data support the use of the THRβ agonist resmetirom to ameliorate atherogenic dyslipidemia as well as reduce the risk of MACE, cardiovascular and all-cause mortality [16,18,19]. Other agents that have demonstrated improvements in clinical outcomes include incretin mimetics (semaglutide, tirzepatide) and the pan-PPAR agonist lanifibranor.

Semaglutide has been proven to ameliorate HF symptoms, adverse cardiac remodeling, lower blood pressure, improve serum lipid profiles, and, most importantly, reduce the risks of MACE and cardiovascular death [36,37,38,39,40,41,42,43,44,45,46]. Similarly, tirzepatide has been demonstrated to effectively prevent HF worsening, myocardial infarction, stroke, and reduce cardiovascular mortality, alongside improvements in biochemical and clinical parameters [53,54,55,56]. However, these data are largely extrapolated from post hoc analyses of studies in patients with overweight/obesity and/or T2DM and require confirmation in large-scale prospective trials with predefined cardiovascular endpoints in individuals with MASLD/MASH.

Lanifibranor improved dyslipidemia and arterial hypertension and was associated with a reduction in overall cardiovascular risk in a single randomized trial. While a pan-PPAR approach may offer significant hepatic and extrahepatic benefits to individuals with MASLD/MASH, the possible adverse effects of selective PPAR and dual-PPAR-α/γ activation must be taken into consideration. Several dual-PPAR-α/γ agonists have been abandoned in late-stage clinical trials because they paradoxically aggravated congestive HF and kidney disease in T2DM patients [198,199]. Therefore, a careful benefit-risk assessment of pan-PPAR drug candidates is warranted in individuals with MASLD/MASH and high cardiovascular risk.

Multiple investigational compounds, including FXR agonists, MPC inhibitors, DGAT2 inhibitors, FGF21 analogs, BUDCA, and icosabutate, have been found to improve serum lipid profiles and markers of glycemic control. While certainly positive, these effects alone are insufficient to establish definitive benefit in terms of symptoms, quality of life, disease progression, prognosis, and the risks of cardiovascular events, hospitalization, and death [200,201]. Whether these changes would possibly translate into meaningful reductions in hard clinical endpoints remains to be confirmed in further clinical trials. Given the bidirectional relationship between MASLD and cardiometabolic risk factors, cardiovascular outcomes should be prioritized in further clinical research.

Moreover, drug development for MASLD in general remains a major challenge due to the biological and clinical heterogeneity of the disease itself, the impact of comorbidities, limitations of available endpoints, high variability in trial duration, placebo response, and endpoint assessment [202,203]. Considering the complex pathogenesis of MASLD and its comorbidities, combination therapies utilizing two or more agents that target different pathogenetic links may represent a rational alternative therapeutic approach. Emerging frameworks for personalized MASLD care integrate lifestyle modification, weight management and metabolic therapies as well as liver-targeted agents [11,50,203].

Liver- and metabolism-directed combination therapy represents a viable future strategy to optimize treatment efficiency and safety in MASLD/MASH, especially in patients with concomitant CVD. Investigational drug classes that have demonstrated notable potential for therapeutic synergism in MASLD include THRβ agonists, incretin mimetics, MPC inhibitors, and DGAT2 inhibitors. Available evidence suggests potential synergism between THRβ and FXR agonists; incretin mimetics, lipogenesis inhibitors, and/or SGLT2 inhibitors; incretin mimetics, GPR119 agonists, and/or FGF21 analogs; SPPARMα and SGLT2 inhibitors, etc. [11,50]. In addition, development of unimolecular poly-agonists targeting multiple receptors or pathways simultaneously is considered a viable strategy to optimize treatment efficacy, safety, and compliance [31]. Moreover, siRNA- and antisense oligonucleotide-based personalized therapies may represent a new frontier in the management of MASLD and associated comorbidities [204].

## Figures and Tables

**Table 1:** Comparative characteristics of the main drug candidate classes and their cardiovascular effects in MASLD/MASH.

Class	Mechanism of Action	Compounds	Key Clinical Trials *	Cardiovascular Effects
THRβ agonists	Selective hepatic THRβ activation increases fatty acid oxidation and lipid turnover, reduces steatosis, promotes mitophagy, and alleviates mitochondrial dysfunction	Resmetirom **	Phase 2b: 1 RCT (*n* = 125) [190] Phase 3: 2 RCT (*n* = 972, *n* = 966) [191,192] 1 matched cohort trial (*n* = 2380) [19] 2 meta-analyses: 4 RCT (*n* = 2183) [16]; 5 RCT (*n* = 1959) [17]	↓ TG, LDL-C, Lp(a), Apo-B, Apo-C3, small dense low-density lipoproteins, large very low-density lipoproteins, chylomicrons ↑ Adiponectin ↓ MACE risk, all-cause mortality
		TERN-501	Phase 2a: 1 RCT (*n* = 162) [22]	↓ TG, VLDL-C, Lp(a)
		VK2809	Phase 2b: 1 RCT (*n* = 248) [23]	↓ TG, LDL-C, Lp(a), Apo-B, Apo-C3
		ASC41	Phase 2: 1 RCT *** [24,25]	↓ TG, TC, LDL-C
Incretin mimetics	GLP1R/GIPR/GCCR activation enhances insulin secretion, inhibits gluconeogenesis and lipogenesis, promotes lipolysis and fatty acid oxidation, ameliorates endothelial dysfunction, reduces vascular resistance, optimizes myocardial energy metabolism, reduces systemic inflammation and cardiac oxidative stress	Semaglutide **	Phase 3: 1 RCT in overweight/obesity and atherosclerotic CVD (*n* = 17,604) [41]; 1 RCT in T2DM and CKD (3533) [42]; 1 RCT in obesity and HFpEF (*n* = 529) [37,38]; 1 RCT in obesity, T2DM and HFpEF (*n* = 617) [39] 3 pooled post hoc analyses: 2 RCT in obesity and HFpEF (*n* = 1146) [40]; 2 RCT in obesity and HFpEF [44]; 4 RCT in obesity and/or T2DM (*n* = 22,283) [36] 3 meta-analyses: 4 RCT in overweight/obesity (*n* = 3051) [45]; 6 RCT in overweight/obesity (*n* = 4744) [46]; 29 RCT in T2DM (*n* = 26,985) [193]	↓ TG, TC, LDL-C, VLDL-C ↑ HDL-C ↓ SBP, DBP, HF symptoms ↑ Exercise capacity ↓ Cardiac remodeling ↓ Loop diuretic use and dose ↓ Time to 1st HF event, time to CV death, time to composite of 1st HF event or CV death ↓ Risk of worsening HF events, risk of composite of CV death or HF events, MACE risk
		Tirzepatide	Phase 3: 1 RCT in T2DM and atherosclerotic CVD (*n* = 13,299) [55]; 1 RCT in obesity and HFpEF (*n* = 731) [56] 1 meta-analysis: 8 RCT in T2DM (*n* = 7491) [53];	↓ TG, TC, LDL-C, VLDL-C, Apo-B, Apo-C3 ↑ HDL-C ↓ SBP, DBP, HF symptoms ↓ Risks of worsening HF, myocardial infarction, stroke, CV death
		Pemvidutide	Phase 1: 1 RCT (*n* = 64) [59] Phase 2b: 1 RCT *** (*n* = 212) [60] 1 post hoc analysis: 1 RCT (*n* = 34) [61]	↓ TG ↑ Plasma lipidomics profile ↓ SBP
		Efinopegdutide	Phase 2a: 1 RCT (*n* = 145) [57]	↓ TG, TC, LDL-C
		Survodutide	Phase 2: 1 RCT in obesity (*n* = 387) [63]	↓ SBP, DBP
		Retatrutide	Phase 2a: 1 RCT in obesity (*n* = 338) [64]	↓ TG, TC, LDL-C, VLDL-C ↓ SBP, DBP
GPR119 agonists	GPR119 activation promotes insulin secretion from pancreatic β-cells, GLP1 secretion from enteroendocrine cells, and attenuates steatosis, inflammation, and fibrogenesis in the liver	Vanoglipel	Phase 2a: 1 RCT (*n* = 109) [68]	↓ TG ↑ Plasma lipidomics profile
FXR agonists	Hepatic FXR activation downregulates bile acid synthesis, suppresses de novo lipogenesis via SREBP-1c repression, promotes fatty acid oxidation and lipolysis via PPARα activation	Vonafexor	Phase 2a: 1 RCT (*n* = 120) [73]	↓ TG, TC, LDL-C, VLDL-C, Apo-B
PPAR agonists	PPARα activation promotes fatty acid β-oxidation, lipolysis, and ketogenesis; PPARβ/δ and PPARγ activation improves insulin sensitivity, promotes lipid uptake and utilization, facilitates glucose uptake by adipocytes, enhances adiponectin secretion, and upregulates FGF21	Pemafibrate	Pilot: 1 non-randomized uncontrolled trial in T2DM and hypertriglyceridemia (*n* = 17) [86] Phase 3: 1 RCT in T2DM and hypertriglyceridemia (*n* = 10,497) [84]	↓ TG, VLDL-C, remnant cholesterol, Apo-C3 ↑ Diastolic function
		Lanifibranor	1 post hoc analysis: 1 RCT (*n* = 247) [97]	↓ TG, TC, VLDL-C, Apo-B, Apo-B/Apo-A1 ratio, high-sensitivity C-reactive protein ↓ DBP ↑ HDL-C ↓ cardiovascular risk
		Chiglitazar	Phase 2: 1 RCT in T2DM (*n* = 200) [102] Phase 3: 1 RCT in T2DM (*n* = 533) [103]	↓ TG, TC, free fatty acids ↑ HDL-C
Lipogenesis inhibitors	Inhibition of hepatic de novo lipogenesis and TG synthesis reduces steatosis, oxidative stress, lipotoxicity-driven inflammation, and fibrogenesis	Denifanstat	Phase 2b: 1 RCT (*n* = 168) [110]	↓ LDL-C, saturated TG
		PXL065	Phase 2: 1 RCT (*n* = 117) [121]	↑ Adiponectin
FGF21 analogs	Activation of FGF21 receptors enhances insulin sensitivity, facilitates glucose utilization and fatty acid oxidation, suppresses de novo lipogenesis, and increases energy expenditure	Pegozafermin	Phase 2b: 1 RCT (*n* = 222) [135] 1 network meta-analysis: 8 RCT (*n* = 963) [139]	↑ Serum lipid profile
		Efruxifermin	Phase 2a: 1 RCT (*n* = 80) [194] Phase 2b: 3 RCT (*n* =128, *n* = 31, *n* = 181) [195,196,197] 2 meta-analyses: 4 RCT (*n* = 450) [136]; 8 RCT (*n* = 963) [139]	↓ TG, TC, LDL-C, Apo-B, Apo-C3 ↑ HDL-C, adiponectin
		Efimosfermin alfa	Phase 2: 1 RCT + open-label extension (*n* = 65) [138] 1 network meta-analysis: 8 RCT (*n* = 963) [139]	↓ TG, TC, LDL-C ↑ HDL-C, adiponectin

* Patient populations are MASLD/MASH unless specified otherwise; ** approved by the FDA; *** trial ongoing; ↓, decrease; ↑, increase; Apo, apolipoprotein; CV, cardiovascular; CVD, cardiovascular disease; DBP, diastolic blood pressure; FGF21, fibroblast growth factor 21; FXR, farnesoid X receptor; GCCR, glucagon receptor; GIPR, glucose-dependent insulinotropic peptide receptor; GLP1, glucagon-like peptide-1; GLP1R, glucagon-like peptide-1 receptor; GPR119, G protein-coupled receptor 119; HDL-C, high-density lipoprotein cholesterol; HF, heart failure; HFpEF, heart failure with preserved ejection fraction; LDL-C, low-density lipoprotein cholesterol; Lp(a), lipoprotein (a); MACE, major adverse cardiovascular events; PPAR, peroxisome proliferator-activated receptor; RCT, randomized clinical trial; SBP, systolic blood pressure; SREBP-1c, sterol regulatory element-binding protein 1; T2DM, type 2 diabetes mellitus; TC, total cholesterol; TG, triglycerides; THRβ, thyroid hormone receptor β; VLDL-C, very low-density lipoprotein cholesterol.

## Data Availability

No new data were created or analyzed in this study. Data sharing is not applicable to this article.

## Abbreviations

The following abbreviations are used in this manuscript:

A3AR	A3 adenosine receptor
ACC	Acetyl-coenzyme A carboxylase
Apo	Apolipoprotein
BID	Twice daily
BUDCA	Berberine ursodeoxycholate
CCR	CC-motif chemokine receptor
CoA	Coenzyme A
CVD	Cardiovascular disease
DGAT	Acylcoenzyme A:diacylglycerol acyltransferase
FASN	Fatty acid synthase
FDA	US Food & Drug Administration
FFAR	Free fatty acid receptor
FGF	Fibroblast growth factor
FXR	Farnesoid X receptor
GCGR	Glucagon receptor
GIP	Glucose-dependent insulinotropic peptide
GIPR	Glucose-dependent insulinotropic peptide receptor
GLP1	Glucagon-like peptide-1
GLP1R	Glucagon-like peptide-1 receptor
GLP1RA	Glucagon-like peptide-1 receptor agonist
GPR119	G protein-coupled receptor 119
HbA_1C_	Glycated hemoglobin
HDL-C	High-density lipoprotein cholesterol
HF	Heart failure
HFpEF	Heart failure with preserved ejection fraction
HOMA-IR	Homeostatic model assessment for insulin resistance
Lp(a)	Lipoprotein(a)
MACE	Major adverse cardiovascular events
MASH	Metabolic dysfunction-associated steatohepatitis
MASLD	Metabolic dysfunction-associated steatotic liver disease
MPC	Mitochondrial pyruvate carrier
mRNA	Messenger ribonucleic acid
NLRP3	NLR family pyrin domain-containing 3
PBC	Primary biliary cholangitis
PNPLA3	Patatin-like phospholipase domain-containing protein 3
PPAR	Peroxisome proliferator-activated receptor
PUFA	Polyunsaturated fatty acid
QD	Once daily
QW	Once weekly
RNA	Ribonucleic acid
RORα	Retinoid-related orphan receptor α
SCD1	Stearoyl-coenzyme A desaturase 1
SGLT2	Sodium/glucose cotransporter 2
siRNA	Small interfering ribonucleic acid
SPPARMα	Selective peroxisome proliferator-activated receptor α modulator
SSAO	Semicarbazide-sensitive amine oxidase
T2DM	Type 2 diabetes mellitus
TC	Total cholesterol
TG	Triglyceride
TGFβ1	Transforming growth factor β1
THRβ	Thyroid hormone receptor β
UDCA	Ursodeoxycholic acid
VLDL-C	Very low-density lipoprotein cholesterol
